# Automated Global Longitudinal Strain Assessment in Long-Term Survivors of Childhood Acute Lymphoblastic Leukemia

**DOI:** 10.3390/cancers14061513

**Published:** 2022-03-15

**Authors:** Rafael Gonzalez-Manzanares, Juan C. Castillo, Jose R. Molina, Martin Ruiz-Ortiz, Dolores Mesa, Soledad Ojeda, Manuel Anguita, Manuel Pan

**Affiliations:** 1 Cardiology Department, Reina Sofia University Hospital, 14004 Cordoba, Spain; juanc.castillo.dominguez.sspa@juntadeandalucia.es (J.C.C.); maruor@gmail.com (M.R.-O.); loladoctora@gmail.com (D.M.); soledad.ojeda18@gmail.com (S.O.); manuelanguita@secardiologia.es (M.A.); manuelpanalvarez@gmail.com (M.P.); 2 Maimonides Institute for Research in Biomedicine of Cordoba (IMIBIC), 14004 Cordoba, Spain; josehristomoli@yahoo.es; 3 Hematology Department, Reina Sofia University Hospital, 14004 Cordoba, Spain; 4 Faculty of Medicine and Nursing, University of Cordoba, 14004 Cordoba, Spain

**Keywords:** cardio-oncology, cardiotoxicity, global longitudinal strain, echocardiography, childhood cancer survivor

## Abstract

**Simple Summary:**

Heart failure is a major problem that affects childhood cancer survivors. Thus, early detection of cardiotoxicity before the onset of symptoms is imperative. Global longitudinal strain (GLS) is an echocardiographic tool that can be used to detect subclinical changes in cardiac function. However, its utility in long-term cardiac monitoring is unclear, and its application in routine practice is limited in this setting. We aimed to assess the prevalence of cardiotoxicity in 90 long-term childhood leukemia survivors (CLSs) using conventional echocardiography and automated software that simplifies GLS measurement. Additionally, we compared these measurements and biomarkers with a control group made up of 58 healthy siblings. Our results show that automated GLS outperforms conventional echocardiography in the early detection of cardiotoxicity, emerging as a promising tool in the long-term cardiac surveillance of CLSs.

**Abstract:**

There is limited evidence that supports the use of the global longitudinal strain (GLS) in long-term cardiac monitoring of childhood acute lymphoblastic leukemia survivors (CLSs). Our aim was to assess the utility of automated GLS to detect left ventricular systolic dysfunction (LVSD) in long-term CLSs. Asymptomatic and subclinical LVSD were defined as LVEF < 50% and GLS < 18.5%, respectively. Echocardiographic measurements and biomarkers were compared with a control group. Inverse probability weighting was used to reduce confounding. Regression models were used to identify factors associated with LVEF and GLS in the survivors. Ninety survivors with a median follow-up of 18 (11–26) years were included. The prevalence of LVSD was higher using GLS than with LVEF (26.6% vs. 12.2%). The measurements were both reduced as compared with the controls (*p* < 0.001). There were no differences in diastolic parameters and NT-ProBNP. Survivors were more likely to have Hs-cTnI levels above the detection limit (40% vs. 17.2%, *p* = 0.006). The dose of anthracycline was associated with LVEF but not with GLS in the survivors. Biomarkers were not associated with GLS or LVEF. In conclusion, LVSD detection using automated GLS was higher than with LVEF in long-term CLSs. Its incorporation into clinical routine practice may improve the surveillance of these patients.

## 1. Introduction

Acute leukemia is the most common type of cancer diagnosed during childhood, with acute lymphoblastic leukemia (ALL) being the most common subtype [1]. Since the introduction of anthracycline-based regimens, there has been a remarkable improvement in the outcome of pediatric patients diagnosed with ALL, with contemporary 5-year survival rates being over 90% [2]. Unfortunately, the benefit of anthracyclines comes at the price of an increased risk of cardiac-related problems [3], which constitute the main cause of morbimortality in childhood acute lymphoblastic leukemia survivors (CLSs) along with subsequent malignant neoplasms [4]. The cumulative incidence of symptomatic heart failure (HF) among childhood cancer survivors (CCSs) treated with cardiotoxic drugs increases progressively with time, reaching up to 10.6% at 40 years after diagnosis [5]. Even with low doses of anthracyclines, there is a decline in left ventricular ejection fraction (LVEF) over time [6], and a significant number of CCSs develop asymptomatic left ventricular systolic dysfunction (LVSD) (6–8% at 9 to 23 years from diagnosis) long before HF onset [7]. Within this framework of growing numbers of CCSs with a well-known risk of developing LSVD and subsequent HF, the investigation of early markers of cardiotoxicity is of paramount importance [8]. Despite the fact that international guidelines [9,10] recommend periodic cardiac screening in long-term cancer survivors, there is no general agreement regarding the most appropriate surveillance techniques in this setting [11].

Global longitudinal strain (GLS), an echocardiographic measurement of myocardial deformation, has received increasing attention with regard to the monitoring of ongoing cardiotoxic treatments [12], as its reduction precedes LVEF decline [13,14], allowing the detection of subclinical LVSD. However, data are limited on the prevalence of subclinical LVSD in CCSs and the role of GLS in survivorship cardiac surveillance [15,16,17]. Its progressive incorporation into clinical routine practice and the development of automated strain software [18] have set the stage for GLS to be considered a promising tool in long-term cardiac monitoring. We hypothesized that LV assessment with GLS would provide added value to the detection of cardiotoxicity in CLSs.

The purpose of this study was to determine the prevalence of asymptomatic and subclinical LVSD in long-term CLSs by means of LVEF and automated GLS, respectively. Furthermore, we aimed to compare echocardiographic parameters and biomarkers with a control group made up of healthy siblings.

## 2. Materials and Methods

### 2.1. Participants

The current work was conducted within the framework of the CTOXALL study (Long-term Cardiotoxicity in Childhood Acute Lymphoblastic Leukemia Survivors Exposed to Anthracycline Therapy). CTOXALL is a cross-sectional study of a retrospective, single-center cohort of patients diagnosed with ALL before 18 years of age between 1985 and 2015 and a control group made up of healthy siblings. The CTOXALL study aims to describe the prevalence of long-term cardiotoxicity in CLSs and to investigate novel echocardiographic parameters, biomarkers, and genetic variants that permit the detection of subclinical cardiotoxicity and the identification of high-risk patients who require closer cardiac monitoring. The present study sought to assess left ventricle abnormalities by adding GLS to conventional echocardiographic parameters. Additionally, 3D-LVEF and cardiac biomarkers were evaluated. The CTOXALL study protocol was approved by the Local Clinical Research Ethics Committee according to institutional and Good Clinical Practice guidelines. Written informed consent was obtained from all participants/parents/legal guardians.

Participants were recruited from May 2019 to January 2022 at Reina Sofia University Hospital (Córdoba, Spain). Patients were eligible if they were aged <18 years at ALL diagnosis and had a minimum of 3 years of follow-up after the last anthracycline dose at inclusion. An individual with a congenital heart disease (ventricular septal defect) was excluded. Additionally, a sample of siblings of survivors willing to participate were recruited as a comparison group.

### 2.2. Clinical Assessment

A complete clinical assessment was conducted, which included assessment of the patients’ histories and physical examinations. Clinical data were collected from medical records. The anthracycline cumulative doses were converted to doxorubicin equivalents using previously described conversion factors [19]: 0.6 for daunorubicin, 0.8 for epirubicin, and 10.5 for mitoxantrone. Chest radiotherapy was considered when the heart region was involved, including total-body irradiation.

### 2.3. Echocardiography

All studies were performed using the same echocardiographic systems (EPIQ CVx and iE33, Philips Medical Systems, Andover, MA, USA) by qualified echocardiographers. Standard echocardiographic parameters were obtained according to current recommendations [20,21]. LVEF and GLS measurements were performed in random order and without knowledge of each participant’s status. When available and feasible, 3D-LVEF was calculated using machine-learning-based, automated dynamic quantification software (Philips Dynamic Heart Model) [22]. LV dysfunction was defined as EF < 50%, in line with previous long-term cardiotoxicity studies and recent Cardio-Oncology Society recommendations [23]. GLS was measured from the apical two-, three-, and four-chamber views using a semiautomated assessment with AutoStrain (TomTec-Arena, TomTec Imaging Systems, Unterschleissheim, Germany). After automatic calculation using the software, the operator manually adjusted endocardial borders if needed [18]. Subclinical LV dysfunction was defined as abnormal GLS. For simplicity, GLS measurements are reported as their absolute values. We considered GLS < 18.5% to be abnormal based on the lower limit of the 95% confidence interval in the control group.

### 2.4. Variability Analysis

To test the intraobserver and interobserver variability, 20 echocardiograms were randomly selected, and LVEF and GLS were measured by the same investigator who performed the analysis and a second investigator, respectively.

### 2.5. Laboratory Tests

Venous blood samples for biomarkers and complete blood count were obtained and collected in Vacutainer tubes with EDTA and tubes with no anticoagulant at the time of echocardiography. N-terminal prohormone of brain natriuretic peptide (NT-ProBNP) and high-sensitivity cardiac troponin I (Hs-cTnI) were quantified from serum samples using an Atellica Immunoassay Analyzer (Siemens Healthineers, Walpole, MA, USA). The cut-off value of NT-proBNP was >125 pg/mL. Sex-specific Hs-cTnI cut-off values were based on manufacturer recommendations considering the 99th percentile of values in the healthy population in our area: >37 ng/L for females and >56 ng/L for males.

### 2.6. Statistical Analysis

Categorical variables are presented as counts (percentages), and continuous variables are summarized as means ± standard deviations or medians (interquartile ranges) according to their distribution, which was assessed using the Shapiro–Wilk test and QQ plots. Survivors and controls were compared using the chi-square test or Fisher’s exact test for categorical data and Student’s T-test or the Mann–Whitney U-test for continuous data, as appropriate.

The prevalence of asymptomatic and subclinical LV dysfunction was calculated in CLSs and controls, as defined by values of LVEF < 50% and GLS < 18.5%, respectively.

Linear regression was used to compare echocardiographic measurements and biomarkers between groups. Inverse probability of treatment weighting (IPW) was used to account for differences between the two groups [24]. The propensity score was estimated using logistic regression with sex, age, body mass index, heart rate, and diastolic blood pressure as covariates. Standardized mean differences before and after the weighting were used to evaluate the balance of the groups regarding the covariates. A difference of <10% was considered to indicate good balance. The distribution of the propensity score before and after the weighting was plotted to assess the degree of overlap between the two groups. Standard errors of the IPW linear regression coefficients were obtained using robust sandwich-type variance estimators [25].

Multivariable linear regression analyses were conducted to determine significant predictors for LVEF and GLS in survivors. Covariates included: sex, age, age at diagnosis, time since diagnosis, BMI, heart rate, systolic and diastolic blood pressure, hypertension, diabetes mellitus, hypercholesterolemia, obesity, hypothyroidism, smoking, sedentarism, cumulative anthracycline dosage, anthracycline dose >250 mg/m^2^, radiotherapy, and hematopoietic stem-cell transplantation (HSCT). The final regression models considered multicollinearity and contained all of the variables that were considered to be clinically meaningful and those showing a *p*-value in the univariable analysis <0.2. To account for missing values, multiple imputations were performed. The frequency of missing values ranged from 0% to 5%, and they were considered to be missing at random.

Intraobserver and interobserver agreement was assessed using ICC and the Bland–Altman method, in which the difference between the two paired measurements (*y* axis) was plotted against the mean of the two measurements (*x* axis) [26]. The limit of agreement with 95% CI was computed as the mean difference ± 1.96 SD.

Statistical analyses were performed using the SPSS software (version 24; IBM Corp., Armonk, NY, USA) and R software (version 4.0.3; R Foundation for Statistical Computing, Vienna, Austria).

## 3. Results

### 3.1. Participant Characteristics

**Figure 1:**
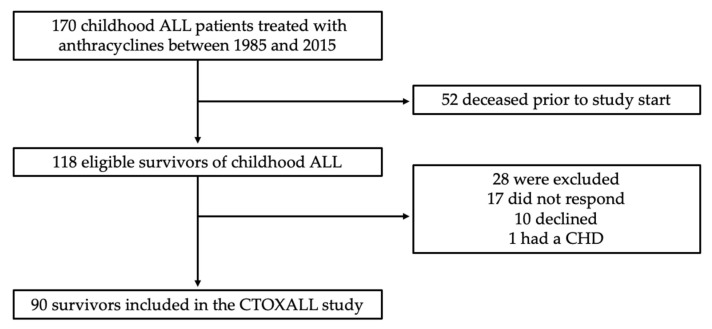
Flow diagram of the childhood leukemia survivors included in the analysis. ALL, acute lymphoblastic leukemia; CHD, congenital heart defect.

A total of 170 patients aged <18 years were diagnosed with ALL during the specified period. Among these, 52 patients died prior to the study’s start date and among the 118 eligible patients, 28 patients were not included: 17 patients did not respond to the invitation, 10 patients refused to participate, and 1 patient had a CHD (Figure 1).

In all, 90 CLSs were included in the current study. The median age at diagnosis was 4 (3–7) years old, and the mean age at recruitment was 24.6 ± 9.7 years old. The median time from diagnosis was 18 (11–26) years, and the median isotoxic cumulative anthracycline dose was 138 (72–192) mg/m^2^. Three patients (3.3%) required cardiac irradiation, and 17 patients (18.9%) underwent HSCT. A total of 58 healthy siblings were included as the control group. The characteristics of the survivors and controls, cardiovascular risk factors, and ALL treatments are shown in Table 1. The controls were similar in age, body measurements, and the prevalence of cardiovascular risk factors but were more likely to be female (*p* = 0.018) and less likely to have hypothyroidism (*p* = 0.043). High-density lipoprotein (HDL) levels were lower (*p* = 0.006) and triglyceride levels were higher in the survivors (*p* = 0.045). Regarding biomarkers, there were no differences in NT-ProBNP levels, but Hs-cTnI levels were higher in the survivors (*p* = 0.032).

### 3.2. Prevalence of Left Ventricular Systolic Dysfunction and Biomarkers Abnormalities

**Figure 2:**
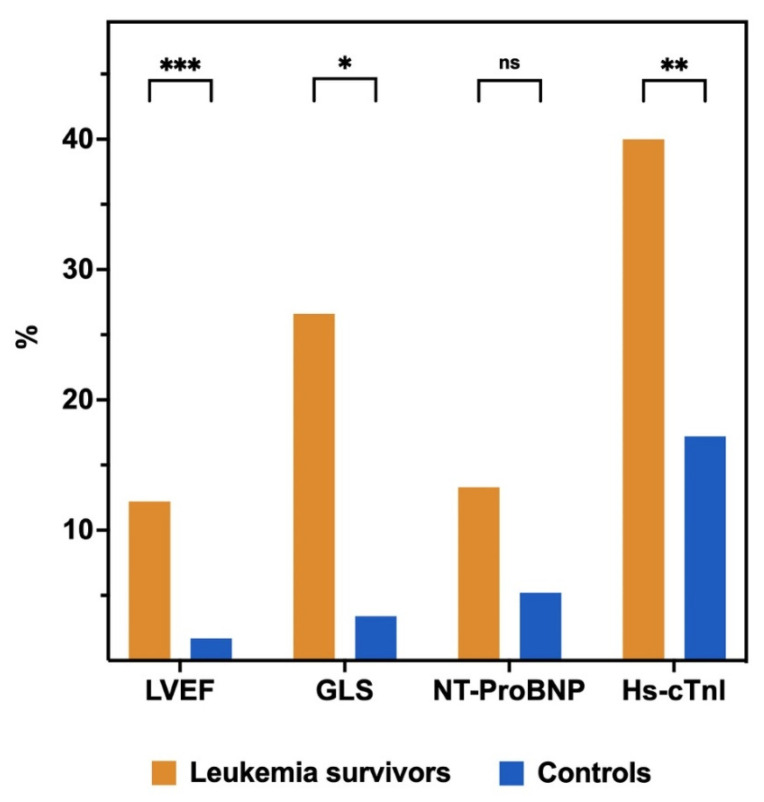
Prevalence of left ventricular systolic dysfunction and biomarkers abnormalities in leukemia survivors and controls. Cut-off values were LVEF < 50%, GLS < 18.5%, NT-ProBNP > 125 pg/mL, and Hs-cTnI > 2.5 ng/L. * *p* < 0.001, ** *p* < 0.01, *** *p* < 0.05. ns, Not significant.

2D-LVEF was available and feasible in all patients, automated 3D-LVEF was available in 62.2% of patients, and feasible in 91.4% of them. Automated GLS was available in all patients and feasible in 96% of patients. The prevalence of asymptomatic LVSD was significantly higher in the survivor group: 12.2% vs. 1.7%, *p* = 0.029. The prevalence in ALL survivors using automated 3D-LVEF was 11.6%; only one survivor had an LVEF < 40%. The prevalence of subclinical LVSD was also higher in the survivors: 26.6% vs. 3.4%, *p* = 0.001. There were no significant differences in the proportion of patients with NT-ProBNP and Hs-cTnI levels above the upper normal limit. However, more frequently, survivors had Hs-cTnI above the detection limit of 2.5 ng/L (40% vs. 17.2%, *p* = 0.006) (Figure 2).

### 3.3. Comparison of Echocardiographic Parameters between Groups

**Figure 3:**
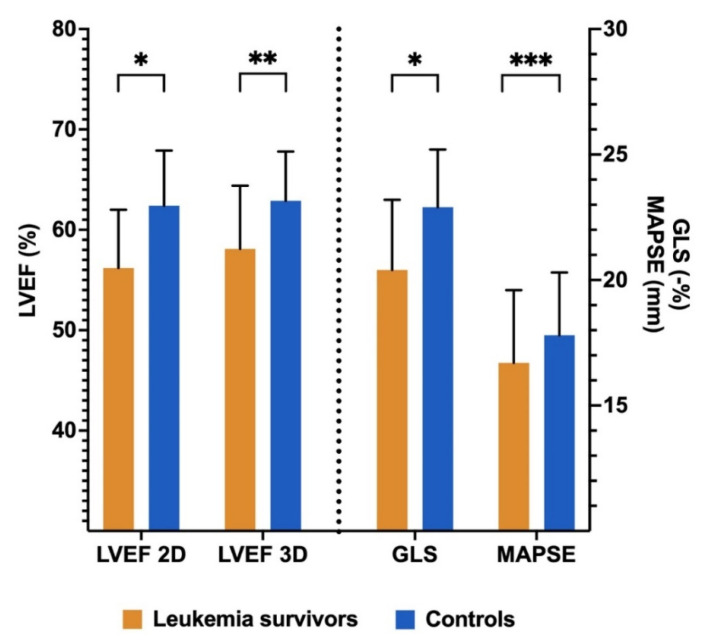
Left ventricular systolic function parameters in leukemia survivors and controls. LVEF, left ventricular ejection fraction; GLS, global longitudinal strain; MAPSE, mitral annular plane systolic excursion, average of septum, and lateral. * *p* < 0.001, ** *p* < 0.01, *** *p* < 0.05.

Table 2 shows left ventricular structure and function echocardiographic measurements of the ALL survivors and the control subjects. The average left ventricular systolic parameters of the survivors were all within the normal range but were lower as compared with control subjects before and after IPW: LVEF Teichholz (66.8 ± 6.4 vs. 72.2 ± 7.6, *p* < 0.001), LVEF 2D (56.2 ± 5.8 vs. 62.4 ± 5.5, *p* < 0.001), 3D-LVEF (58.1 ± 6.3 vs. 62.9 ± 4.9, *p* = 0.003), GLS (-%) (20.4 ± 2.8 vs. 22.9 ± 2.3, *p* < 0.001), and MAPSE (16.7 ± 2.9 vs. 17.8 ± 2.5, *p* = 0.019) (Figure 3). Left ventricular morphological and diastolic measurements were within the normal range, and there were no differences between groups. The variables used in the IPW, the standardized mean differences, and the Propensity Score distributions of the unweighted and weighted samples are represented in Supplementary Figure S1. PSM resulted in an excellent balance of covariates with standardized mean differences ≤10% on all the variables included in the Propensity Score.

### 3.4. Predictors of Left Ventricular Ejection Fraction in Leukemia Survivors

Univariable and multivariable linear regression models are shown in Table 3. The predictors of LVEF in the univariable analysis were time since diagnosis, hypertension, diabetes mellitus, anthracycline dose, radiotherapy, HSCT, and Hs-cTnI. In the multivariable model, independent predictors of LVEF were diabetes mellitus, anthracycline dose >250 mg/m^2^, and radiotherapy.

### 3.5. Predictors of Global Longitudinal Strain in Leukemia Survivors

Univariable and multivariable linear regression models are shown in Table 4. The predictors of GLS in the univariable analysis were age at exam, smoking, and HSCT. In the multivariable model, only smoking and HSCT were independent predictors of GLS. There was a borderline significant association with sex.

### 3.6. Intraobserver and Interobserver Variability Analysis

The intraclass correlation coefficient (ICC) for intraobserver agreement was 0.93 (95% CI 0.84 to 0.97) for LVEF and 0.95 (95% CI 0.87 to 0.98) for GLS. The ICC for interobserver agreement was 0.89 (95% CI 0.73 to 0.95) for LVEF and 0.92 (95% CI 0.82 to 0.97) for GLS. Bland–Altman plots are shown in Supplementary Figure S2.

## 4. Discussion

The main findings of the present study can be summarized as follows: (a) The prevalence of LVSD detected using GLS was higher than with LVEF among long-term CLSs. (b) The LV systolic parameters were decreased as compared with the control group. (c) NT-ProBNP and Hs-cTnI were not predictors of LVEF or GLS in ALL survivors. (d) Novel echocardiographic tools such as automated 3D-LVEF and automated GLS were useful in the long-term cardiac monitoring of these patients.

Despite the fact that 2D-LVEF continues to be the most frequently used tool for monitoring cardiotoxicity, novel and more reproducible techniques such as GLS, 3D-LVEF, and cardiac magnetic resonance (CMR) are gaining popularity. CMR is the gold standard for LVEF quantification and allows for tissue characterization, but its use is limited due to reduced availability and high costs [27]. The main role of GLS in contemporary cardio-oncology practice is the early detection of cardiotoxicity during ongoing cancer therapies [28]. However, its value in clinical decision making is not clearly established, and there is no strong evidence that supports a GLS-based approach to the initiation of cardioprotective therapy (angiotensin-converting enzyme inhibitors or beta-blockers) [29]. There are also few data regarding the use of GLS in long-term CCSs, and the few studies carried out in this population have focused on the detection of cardiotoxicity but not on its usefulness for the prediction of HF or on the benefit of cardioprotective therapy initiation in long-term CCSs with GLS impairment.

GLS has been proposed to be a more sensitive tool than LVEF in the detection of cardiotoxicity in long-term CCSs [17,30]. However, GLS is not often measured in clinical practice because of a lack of availability. In the present study, we also found a higher prevalence of LVSD with GLS than with LVEF, but, for the first time, we employed automated software [18] that may have helped to generalize the use of this tool in the cardiac surveillance of CCS. Our finding of a 26.6% prevalence of GLS impairment after a median 18-year follow-up time after diagnosis is consistent with previous data. Christiansen et al. demonstrated an abnormal GLS in 28% of survivors of childhood lymphoma or ALL after a 20-year follow-up time [31]. Armstrong et al. reported a 28% prevalence of impaired GLS in CSS from the St. Jude Lifetime Cohort Study [15]. Contrary to our results, both studies found an association between radiotherapy exposure, high anthracycline doses, and abnormal GLS. This disparity may be explained by a higher percentage of radiotherapy exposure and a stricter definition of high anthracycline dose (>300 mg/m^2^ instead of 250 mg/m^2^). For instance, in the former study, GLS impairment was present in up to 40% of Hodgkin Lymphoma survivors, who were more frequently exposed to radiotherapy (69%) and to higher anthracycline doses, i.e., 160 (50–500) mg/m^2^. In that sense, another explanation would be a lack of power in our study to detect a weaker association between these treatments and GLS as compared with LVEF, as is illustrated in the later study. Conversely, the observed association between HSCT and GLS has recently been examined by Massey et al., who reported a 32.7% prevalence of GLS impairment in a cohort of long-term HSCT survivors [32]. Despite a similar follow-up time to our study, the higher prevalence of abnormal GLS was consistent with the older age of the participants at the time of examination (35 ± 9.7 years) and the fact that all of them were HSCT recipients, while in our study, only a fifth received this treatment. More recently, Niemela et al. described a 11% prevalence of GLS impairment in 90 long-term CCSs exposed to anthracyclines. The lower frequency of subclinical LVSD may be explained by a shorter follow-up (8.1 years post treatment) and a more restrictive GLS cut-off value of 17.5%. In accordance with our results, the dose of anthracycline and radiotherapy were not predictors of GLS in the survivors, suggesting that subclinical LVSD detected with GLS may develop even with low anthracycline doses. Similarly, other studies that have addressed this issue in pediatric CCSs did not observe an independent relationship between GLS and anthracycline dose [33,34].

The prevalence of asymptomatic LVSD using conventional 2D-LVEF (12.2%) and automated 3D-LVEF (11.6%) was similar and comparable to previous reports of long-term CCSs, in which the prevalence ranged from 4% to 16.5% [16,32,35]. Although in the present study the majority of LVSD cases were midrange EF (40–50%), this finding at first echocardiographic screening is an important predictor of LVEF < 40% in long-term CCSs [7]. Initial studies evaluating cardiotoxicity in these patients used less reliable echocardiographic parameters such as fractional shortening and LV wall thinning [36,37,38]. Subsequently, certain studies have included the use of 2D-LVEF [33,35,39] and, more recently, a 3D-LVEF assessment [15,32], which was preferred due to the increased accuracy and reproducibility [21]. As in the case of GLS, its implementation into routine practice is partly hampered, as it requires experience, and it is time-consuming. The use of fully automated 3D software seems to overcome these limitations [22]. As far as we know, no prior studies have evaluated left ventricle function in long-term CCSs by means of automated 3D-LVEF. Regarding predictors of LVEF, we found that well-known cardiotoxicity factors such as chest-directed radiotherapy exposure and high anthracycline doses were associated with LVEF, giving coherence to the abovementioned results [3,40]. Moreover, diabetes mellitus showed the strongest association with LVEF in the multivariable analysis, highlighting the importance of traditional cardiovascular risk factor surveillance in this subset of patients [41], since they have been linked to a remarkable increase in the risk of cardiovascular events among adult-age CCSs [42].

The echocardiographic measurements of the ALL survivors were compared with those of a control group, showing that survivors had significantly reduced measurements when both conventional (MAPSE, Teichholz LVEF, and 2D-LVEF) and modern LV systolic (automated GLS and automated LVEF-3D) assessment tools were used, which is in line with prior studies [17,31]. In contrast, we found no differences in diastolic function parameters, which have also been previously reported [31,43]. We believe that the relatively young age of the survivors at the time of examination (24.6 ± 9.7 years) and the low proportion of patients exposed to radiotherapy (3%), a classic predictor of diastolic dysfunction, may explain this discrepancy. Despite the observed differences in LV systolic parameters, the average measurements in the survivors were within the normal range, and the majority of LVSD cases had an EF within 45–50%. This could explain the absence of symptomatic heart failure cases and of relevant hemodynamic repercussion (E/e’ and NT-ProBNP levels were similar to the control group).

The role of NT-ProBNP and high-sensitivity cardiac troponin in the detection of LVSD in long-term CCSs is limited [44]. Accordingly, we found no association between these biomarkers and LVEF and GLS in the survivors. There were no differences in NT-ProBNP levels between both groups. Conversely, survivors had significantly higher levels of Hs-cTnI, probably reflecting the higher cardiovascular risk of this patients, who also had a more adverse lipid profile, with lower levels of HDL and higher levels of triglycerides. While none of the survivors had an Hs-cTnI level above the p99, 40% of them had values above the detection limit of 2.5 ng/L (while only 17% did in the control group). Low-grade elevations of Hs-cTnI have been strongly associated with cardiovascular outcomes in the general population [45]. Hence, although not a marker of LVSD, Hs-cTnI could help to identify those survivors at a higher risk of cardiovascular events.

The current study has several limitations. First, the cross-sectional design prevented us from identifying the exact moment of the LVSD onset. Second, the conformation of the control group with healthy siblings aimed to control frequently unmeasured and unknown demographic, sociocultural, and environmental factors also resulted in sex differences between the two groups, which could have led to bias. Although inverse probability weighting resulted in a good balance of sex and the other relevant covariates, the presence of residual confounding cannot be completely ruled out. Third, the echocardiographic measurements were not analyzed by a core lab. However, they were performed by an experienced investigator using the same protocol and equipment, the intra- and interobserver variability were good, and there was consistency among different manual and automatic measurements.

## 5. Conclusions

In this study of long-term ALL survivors, the prevalence of LVSD detected using automated GLS software was higher than with LVEF, indicating that subclinical LVSD is common in these patients. As most of them were asymptomatic and NT-ProBNP and Hs-cTnI were not markers of LV systolic impairment, periodic echocardiographic monitoring including GLS seems necessary. The use of automated GLS software may facilitate the incorporation of this tool into routine practice cardiac surveillance of CCSs. Longitudinal data are needed to determine the predictive value of GLS for LVEF deterioration and overt-HF in these patients.

## Figures and Tables

**Table 1:** Characteristics of childhood leukemia survivors and controls.

	CLSs (*n* = 90)	Control Group (*n* = 58)	*p*-Value
Age at diagnosis (years)	4 (3–7)	-	-
Age at exam (years)	24.6 ± 9.7	23.6 ± 10.8	0.593
Time since diagnosis (years)	18 (11–26)	-	-
Sex (% female)	34 (37.8%)	34 (58.6%)	0.018
Weight (kg)	64.8 ± 18.3	61.9 ± 17.2	0.333
Height (cm)	165.6 ± 13.3	164.2 ± 13.8	0.539
Body mass index (kg/m^2^)	23.3 ± 5.1	22.6 ± 4.4	0.346
Body surface area (m^2^)	1.7 ± 0.3	1.7 ± 0.3	0.366
SBP (mmHg)	116.2 ± 11.3	115.6 ± 11.1	0.768
DBP (mmHg)	69.6 ± 7.9	69.4 ± 7.9	0.908
Heart rate (bpm)	72.5 ± 11.1	71.7 ± 11.5	0.646
Current smoker (%)	14 (15.6%)	1 (1.7%)	0.005
Hypertension (%)	3 (3.3%)	0 (0.0%)	0.280
Hypercholesterolemia (%)	12 (13.3%)	4 (6.9%)	0.283
Total cholesterol (mg/dL)	175.6 ± 32.6	174.1 ± 33.8	0.796
HDL (mg/dL)	54.5 ± 15.2	61.4 ± 11.8	0.006
LDL (mg/dL)	95.6 ± 26.6	95.4 ± 27.5	0.971
Triglycerides (mg/dL)	101.9 ± 48.8	86.9 ± 34.2	0.045
HbA1c (%)	5.3 (5.1–5.5)	5.3 (5.1–5.5)	0.690
Diabetes mellitus (%)	4 (4.4%)	1 (1.7%)	0.649
Obesity	9 (10.0%)	7 (12.1%)	0.901
Sedentarism (%)	37 (41.1%)	20 (34.5%)	0.525
Hypothyroidism (%)	7 (7.8%)	0 (0.0%)	0.043
Anthracycline dose (mg/m^2^)	138 (72–192)	-	-
Radiotherapy (%)	3 (3.3%)	-	-
HSCT (%)	17 (18.9%)	-	-
Hs-cTnI (ng/L)	2.5 (2.5–4.5)	2.5 (2.5–2.5)	0.032
NT-ProBNP (pg/mL)	35.0 (35.0–66.5)	35.0 (35.0–49.0)	0.175

CLSs, long-term childhood leukemia survivors; SBP, systolic blood pressure; DBP, diastolic blood pressure; HDL, high-density lipoprotein; LDL, low-density lipoprotein; HbA1c, glycosylated hemoglobin; HSCT, hematopoietic stem-cell transplantation; Hs-cTnI, high-sensitivity cardiac troponin I; NT-ProBNP, N-terminal prohormone of brain natriuretic peptide.

**Table 2:** Echocardiographic parameters of childhood leukemia survivors and controls.

	CLSs (*n* = 90)	Control Group (*n* = 58)	*p*-Value	IPW Beta (RSE), *p*-Value
LVDD (mm)	45.6 ± 6.8	44.9 ± 6.1	0.595	0.09 (1.14), 0.935
LVSD (mm)	28.9 ± 6.0	26.2 ± 4.6	0.005	2.27 (0.92), 0.015
IVS (mm)	7.6 ± 1.5	7.7 ± 1.3	0.824	0.17 (0.23), 0.457
LVEDV (mL)	87.4 ± 29.2	87.1 ± 31.4	0.950	3.19 (5.42), 0.557
LVESV (mL)	39.1 ± 13.9	34.2 ± 15.2	0.046	2.57 (2.65), 0.334
LA volume (mL)	35.8 ± 15.6	36.3 ± 13.8	0.868	2.51 (3.02), 0.407
LVEF Teichholz (%)	66.8 ± 6.4	72.2 ± 7.6	<0.001	5.12 (1.25), <0.001
2D-LVEF (%)	56.2 ± 5.8	62.4 ± 5.5	<0.001	5.45 (0.95), <0.001
3D-LVEF (%)	58.1 ± 6.3	62.9 ± 4.9	<0.001	4.14 (1.33), 0.003
GLS (-%)	20.4 ± 2.8	22.9 ± 2.3	<0.001	2.28 (0.45), <0.001
MAPSE (mm)	16.7 ± 2.9	17.8 ± 2.5	0.016	1.14 (0.48), 0.019
Peak E velocity (cm/s)	94.7 ± 18.4	96.3 ± 15.9	0.579	0.99 (2.87), 0.729
Peak A velocity (cm/s)	57.1 ± 15.6	58.1 ± 17.6	0.714	2.02 (2.85), 0.479
Mitral E/A ratio	1.8 ± 0.7	1.8 ± 0.7	0.785	0.03 (0.11), 0.795
Peak e’ lat velocity (cm/s)	19.4 ± 4.6	20.0 ± 4.4	0.439	0.14 (0.74), 0.847
E/e’ lat ratio	5.0 ± 1.3	5.0 ± 1.3	0.849	0.05 (0.21), 0.788
Peak e’ med velocity (cm/s)	13.9 ± 4.0	14.9 ± 3.6	0.131	0.68 (0.64), 0.288
E/e’ med ratio	7.3 ± 2.3	6.8 ± 2.0	0.175	0.39 (0.36), 0.281
E/e’ average ratio	6.2 ± 1.7	5.9 ± 1.5	0.279	0.19 (0.27), 0.465
Gradient RV-RA (mmHg)	19.2 ± 4.9	16.1 ± 3.9	0.024	3.15 (1.39), 0.027

CLSs, long-term childhood leukemia survivors; IPW, inverse probability weighting; RSE, robust standard error; LVDD, left ventricular diastolic diameter; LVSD, left ventricular systolic diameter; IVS, interventricular septum; LVEDV, left ventricular end-diastolic volume; LVESV, left ventricular end-systolic volume; LA, left atrium; LVEF, left ventricular ejection fraction; GLS, global longitudinal strain; MAPSE, mitral annular plane systolic excursion, average of septum, and lateral; Gradient RV-RA, pressure gradient between right ventricle and right atrium.

**Table 3:** Univariable and multivariable linear regression models for LVEF in survivors.

	Univariable			Multivariable		
	Beta	95% CI	*p*-Value	Beta	95% CI	*p*-Value
Sex (female)	2.42	−0.04 to 4.88	0.054	2.07	−0.05 to 4.19	0.056
Age at diagnosis (years)	−0.02	−0.29 to 0.25	0.866			
Age at exam (years)	−0.12	−0.24 to 0.01	0.056			
Time since diagnosis (years)	−0.15	−0.29 to −0.01	0.034	−0.09	−0.21 to 0.025	0.122
BMI (kg/m^2^)	−0.11	−0.35 to 0.13	0.383			
HR (bpm)	0.07	−0.03 to 0.18	0.180			
SBP (mmHg)	0.06	−0.05 to 0.16	0.303			
DBP (mmHg)	−0.19	−0.39 to 0.18	0.403			
Hypertension	−8.02	−14.59 to −1.44	0.017			
Hypercholesterolemia	−2.94	−6.47 to 0.59	0.102			
Diabetes mellitus	−11.64	−17.02 to −6.26	0.001	−8.15	−13.31 to −2.99	0.002
Obesity	−2.41	−6.44 to 1.62	0.238			
Sedentarism	−0.85	−3.33 to 1.62	0.494			
Current smoker	−1.79	−5.13 to 1.55	0.290			
Hypothyroidism	−3.51	−8.00 to 0.98	0.124			
Anthracycline dose	−0.02	−0.04, −0.01	0.004			
Anthracycline dose >250 mg/m^2^	−8.45	−12.99 to −3.89	0.001	−5.88	−10.07 to −1.69	0.006
Radiotherapy	−10.43	−16.85 to −4.01	0.002	−6.49	−12.48 to −0.51	0.034
HSCT	−4.15	−7.14 to −1.16	0.007			
Hs-cTnI (ng/L)	−3.68	−0.68 to −0.05	0.021			
Hs-cTnI > 2.5 ng/L	−0.11	−2.62 to 2.41	0.933			
NT-ProBNP (pg/mL)	−0.19	−0.04 to 0.01	0.066			
NT-ProBNP > 125 pg/mL	−1.86	−5.45 to 1.72	0.305			

LVEF, Left ventricular ejection fraction; CI, confidence interval; BMI, body mass index; HR, heart rate; SBP, systolic blood pressure; DBP, diastolic blood pressure; HSCT, hematopoietic stem-cell transplantation; Hs-cTnI, high-sensitivity cardiac troponin I; NT-ProBNP, N-terminal prohormone of brain natriuretic peptide.

**Table 4:** Univariable and multivariable linear regression models for GLS in survivors.

	Univariable			Multivariable		
	Beta	95% CI	*p*-Value	Beta	95% CI	*p*-Value
Sex (female)	1.03	−0.19 to 2.24	0.096	1.11	−0.04 to 2.27	0.058
Age at diagnosis (years)	−0.11	−0.24 to 0.02	0.097			
Age at exam (years)	−0.06	−0.12 to −0.01	0.043	−0.02	−0.08 to 0.04	0.465
Time since diagnosis (years)	−0.05	−0.12 to 0.02	0.169			
BMI (kg/m^2^)	−0.09	−0.22 to 0.03	0.131			
HR (bpm)	−0.04	−0.09 to 0.01	0.114			
SBP (mmHg)	0.03	−0.02 to 0.09	0.210			
DBP (mmHg)	0.01	−0.061 to 0.08	0.711			
Hypertension	−0.63	−3.90 to 2.64	0.703			
Hypercholesterolemia	−0.82	−2.68 to 1.05	0.385			
Diabetes mellitus	−2.56	−5.79 to 0.67	0.118			
Obesity	−1.46	−3.64 to 0.71	0.184			
Sedentarism	−0.79	−2.01 to 0.41	0.191			
Current smoker	−2.57	−4.10 to −1.05	0.001	−2.29	−3.84 to −0.74	0.004
Hypothyroidism	−0.89	−3.08 to 1.30	0.421			
Anthracycline dose	−0.01	−0.01 to 0.01	0.892	0.01	−0.01 to 0.01	0.410
Anthracycline dose >250 mg/m^2^	−1.21	−3.55 to 1.14	0.308			
Radiotherapy	0.09	−3.18 to 3.37	0.954			
HSCT	−1.49	−3.00 to 0.02	0.052	−1.63	−3.21 to −0.06	0.042
Hs-cTnI (ng/L)	−0.03	−0.16 to −0.15	0.970			
Hs-cTnI > 2.5 ng/L	0.320	−0.91 to 1.55	0.606			
NT-ProBNP (pg/mL)	0.001	−0.01 to 0.01	0.972			
NT-ProBNP > 125 pg/mL	0.442	−1.30 to 2.18	0.615			

GLS, global longitudinal strain; CI, confidence interval; BMI, body mass index; HR, heart rate; SBP, systolic blood pressure; DBP, diastolic blood pressure; HSCT, hematopoietic stem-cell transplantation; Hs-cTnI, high-sensitivity cardiac troponin I; NT-ProBNP, N-terminal prohormone of brain natriuretic peptide.

## Data Availability

The data presented in this study are available on request from the corresponding author.

## Supplementary Materials

The following supporting information can be downloaded at www.mdpi.com/article/10.3390/cancers14061513/s1, Figure S1: Covariate balance and propensity score distributions before and after weighting, Figure S2: Bland–Altman plots for intraobserver and interobserver agreement.
