# Supplementary figures and images for: Automated Global Longitudinal Strain Assessment in Long-Term Survivors of Childhood Acute Lymphoblastic Leukemia
Source: Cancers (Basel). 2022 Mar 15;14(6):1513. doi: 10.3390/cancers14061513 (PMC8946759; doi:10.3390/cancers14061513)

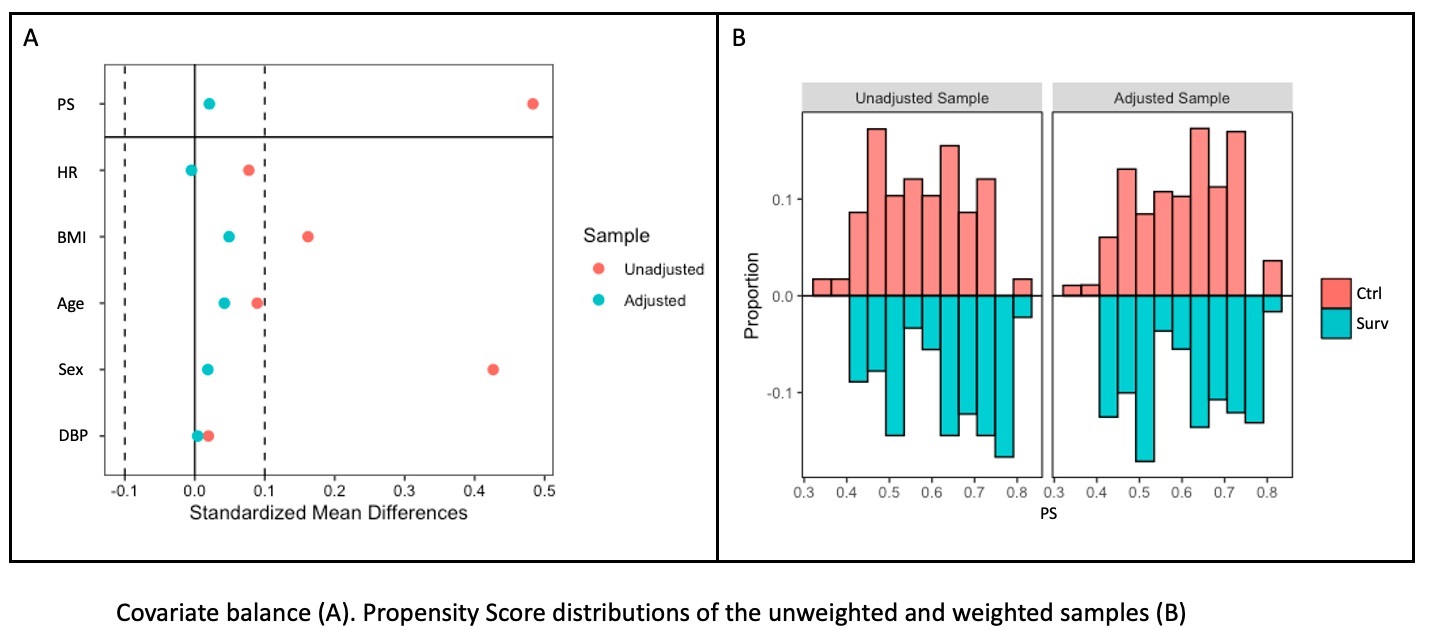

Supplement: Supplementary file 1 [file cancers-14-01513-s001.zip › Supplementary Figure S1.jpg]

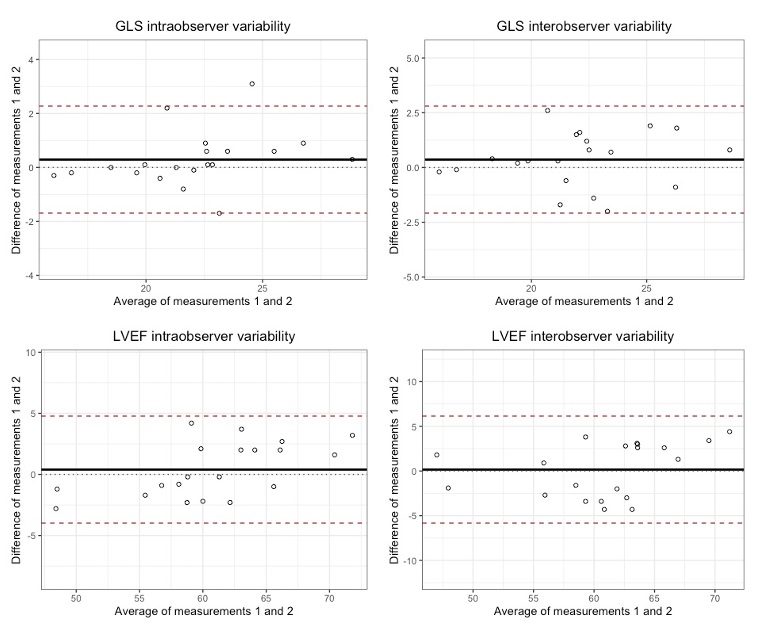

Supplement: Supplementary file 1 [file cancers-14-01513-s001.zip › Supplementary Figure S2.jpg]
